# Pathogenic SLC25A26 variants impair SAH transport activity causing mitochondrial disease

**DOI:** 10.1093/hmg/ddac002

**Published:** 2022-01-13

**Authors:** Florian A Rosenberger, Jia Xin Tang, Kate Sergeant, Marco F Moedas, Charlotte M Zierz, David Moore, Conrad Smith, David Lewis, Nishan Guha, Sila Hopton, Gavin Falkous, Amanda Lam, Angela Pyle, Joanna Poulton, Gráinne S Gorman, Robert W Taylor, Christoph Freyer, Anna Wredenberg

**Affiliations:** Department of Medical Biochemistry and Biophysics, Karolinska Institute, 171 65 Stockholm, Sweden; Faculty of Medical Sciences, Wellcome Centre for Mitochondrial Research, Translational and Clinical Research Institute, Newcastle University, Newcastle upon Tyne NE2 4HH, UK; Oxford Regional Genetics Laboratories, Oxford University Hospitals NHS Foundation Trust, Oxford OX3 7LE, UK; Department of Medical Biochemistry and Biophysics, Karolinska Institute, 171 65 Stockholm, Sweden; Faculty of Medical Sciences, Wellcome Centre for Mitochondrial Research, Translational and Clinical Research Institute, Newcastle University, Newcastle upon Tyne NE2 4HH, UK; Department of Medical Biochemistry and Biophysics, Karolinska Institute, 171 65 Stockholm, Sweden; Oxford Regional Genetics Laboratories, Oxford University Hospitals NHS Foundation Trust, Oxford OX3 7LE, UK; Department of General Medicine, Oxford University Hospitals NHS Foundation Trust, Oxford OX3 9DU, UK; Department of Clinical Biochemistry, Oxford University Hospitals NHS Foundation Trust, Oxford OX3 9DU, UK; Faculty of Medical Sciences, Wellcome Centre for Mitochondrial Research, Translational and Clinical Research Institute, Newcastle University, Newcastle upon Tyne NE2 4HH, UK; NHS Highly Specialised Services for Rare Mitochondrial Disorders, Newcastle upon Tyne Hospitals NHS Foundation Trust, Newcastle upon Tyne NE2 4HH, UK; Faculty of Medical Sciences, Wellcome Centre for Mitochondrial Research, Translational and Clinical Research Institute, Newcastle University, Newcastle upon Tyne NE2 4HH, UK; NHS Highly Specialised Services for Rare Mitochondrial Disorders, Newcastle upon Tyne Hospitals NHS Foundation Trust, Newcastle upon Tyne NE2 4HH, UK; Neurometabolic Unit, Institute of Neurology, Queen Square House, London WC1N 3BG, UK; Faculty of Medical Sciences, Wellcome Centre for Mitochondrial Research, Translational and Clinical Research Institute, Newcastle University, Newcastle upon Tyne NE2 4HH, UK; Nuffield Department of Women’s and Reproductive Health, University of Oxford, Oxford OX3 9DU, UK; Faculty of Medical Sciences, Wellcome Centre for Mitochondrial Research, Translational and Clinical Research Institute, Newcastle University, Newcastle upon Tyne NE2 4HH, UK; NHS Highly Specialised Services for Rare Mitochondrial Disorders, Newcastle upon Tyne Hospitals NHS Foundation Trust, Newcastle upon Tyne NE2 4HH, UK; Faculty of Medical Sciences, Wellcome Centre for Mitochondrial Research, Translational and Clinical Research Institute, Newcastle University, Newcastle upon Tyne NE2 4HH, UK; NHS Highly Specialised Services for Rare Mitochondrial Disorders, Newcastle upon Tyne Hospitals NHS Foundation Trust, Newcastle upon Tyne NE2 4HH, UK; Department of Medical Biochemistry and Biophysics, Karolinska Institute, 171 65 Stockholm, Sweden; Centre for Inherited Metabolic Diseases, Karolinska University Hospital, 171 76 Stockholm, Sweden; Department of Medical Biochemistry and Biophysics, Karolinska Institute, 171 65 Stockholm, Sweden; Centre for Inherited Metabolic Diseases, Karolinska University Hospital, 171 76 Stockholm, Sweden

## Abstract

The *SLC25A26* gene encodes a mitochondrial inner membrane carrier that transports S-adenosylmethionine (SAM) into the mitochondrial matrix in exchange for S-adenosylhomocysteine (SAH). SAM is the predominant methyl-group donor for most cellular methylation processes, of which SAH is produced as a by-product. Pathogenic, biallelic *SLC25A26* variants are a recognized cause of mitochondrial disease in children, with a severe neonatal onset caused by decreased SAM transport activity. Here, we describe two, unrelated adult cases, one of whom presented with recurrent episodes of severe abdominal pain and metabolic decompensation with lactic acidosis. Both patients had exercise intolerance and mitochondrial myopathy associated with biallelic variants in *SLC25A26*, which led to marked respiratory chain deficiencies and mitochondrial histopathological abnormalities in skeletal muscle that are comparable to those previously described in early-onset cases. We demonstrate using both mouse and fruit fly models that impairment of SAH, rather than SAM, transport across the mitochondrial membrane is likely the cause of this milder, late-onset phenotype. Our findings associate a novel pathomechanism with a known disease-causing protein and highlight the quests of precision medicine in optimizing diagnosis, therapeutic intervention and prognosis.

## Introduction

Mitochondria form a central hub in cellular metabolism, with numerous metabolic and biosynthetic pathways entering the organelle. However, metabolic access to mitochondria is highly regulated via a complex network of import machineries, transporters and translocators. The human genome encodes 53 members of the mitochondrial solute carrier family 25 (SLC25), a large group of membrane-embedded proteins that are mostly localized in the highly impermeable inner mitochondrial membrane. These are responsible for the import of amino acids, carboxylic acids, fatty acids, cofactors, inorganic ions and nucleotides across the mitochondrial inner membrane, thereby connecting cytosolic and matrix metabolic functions (1,2). Structurally, the SLC25 family is highly conserved, consisting of a barrel-shaped structure with three pseudo-symmetric domains forming a pore inside the inner mitochondrial membrane. Most act as antiporters, exchanging chemically similar substrates across the lipid bilayer, but uniporter and symporter activities have also been reported. Pathogenic variants in several SLC25 members have been associated with inborn errors of metabolism that are often characterized by discrete forms of encephalopathy, myopathy and neuropathy (3,4).

Given their diverse substrate specificity, defects in members of the SLC25 family manifest with disparate symptoms, ranging from severe, early onset, multi-organ involvement, to mild, late onset, single organ/tissue-specific diseases. This is also true for different pathogenic variants in the same translocator. For instance, pathogenic variants in the predominantly heart- and skeletal muscle-expressed adenine nucleotide translocator, AAC1, encoded by *SLC25A4*, have been reported to cause late-onset dominant progressive external ophthalmoplegia 2 (AdPEO2) associated with the accumulation of quantitative mitochondrial DNA (mtDNA) deletions (OMIM 609283) (5), recessive mtDNA depletion syndrome type 12B (OMIM 615418) (6,7) or the *de novo* dominant mtDNA depletion syndrome 12A (OMIM 103220), defined by early-onset mitochondrial myopathy/cardiomyopathy and associated with marked mtDNA copy number loss and dysfunctional oxidative phosphorylation (OXPHOS) (8,9). The mitochondrial aspartate/glutamate carriers, AGC1 (aralar) and AGC2 (citrin), encoded by *SLC25A12* and *SLC25A13*, respectively, show tissue-specific expression with pathogenic *SLC25A12* variants reported to cause arrested psychomotor development, hypotonia, seizures and global hypomyelination (OMIM 603667) (10,11) or, in the case of *SLC25A13*, neonatal and/or adult-onset type-II citrullinemia (OMIM 603859) (12,13).

The mitochondrial SAM carrier (SAMC) encoded by *SLC25A26* shuttles the primary methyl-group donor S-adenosylmethionine (SAM) across the inner membrane in exchange for S-adenosylhomocysteine (SAH), a by-product of methylation reactions (14). Three families with biallelic pathogenic variants in the *SLC25A26* gene have previously been reported causing neonatal onset of lactic acidosis and childhood episodes of acute cardiopulmonary failure and progressive muscle weakness, with one fatal case of neonatal mortality by respiratory insufficiency and multi-organ failure (15). SAM is a highly abundant enzymatic co-factor within cells and second only to ATP in usage (16). It is synthesized in the cytosol from methionine and ATP in the methionine cycle (17), serving as the methyl group donor for an estimated 200 methyltransferases to methylate nucleic acids, proteins, phosphatidylcholine and carbohydrates, as well as a range of small metabolites (18,19). In mitochondria, SAM-dependent methylation has been demonstrated for mitochondrial RNAs (20–25), as well as NDUFS2, a structural component of complex I (26), the electron transfer flavoprotein ETFβ (27), citrate synthase (28) and the adenosine nucleotide transporters ANT (29). Additionally, the biosynthesis of the lipophilic electron carrier ubiquinone (coenzyme Q, CoQ, Q10) requires several methylation steps in mitochondria (30,31), while pyruvate dehydrogenase (PDH) and α-ketoglutarate dehydrogenase (α-KGDH) require mitochondrial SAM-dependent lipoic acid as co-factor (32). SAMC is the only entry route for SAM into the mitochondrial matrix. The protein is required for survival in *Drosophila melanogaster* (*Dm*) and mice (33), while in humans, pathogenic variants in SAMC resulted in a complex biochemical phenotype, including defects in CoQ and lipoic acid synthesis, OXPHOS dysfunction and mitochondrial translation defects (15).

Here, we report biallelic *SLC25A26* variants in two unrelated cases with disease onset in adult life, who present with biochemical abnormalities comparable to the childhood onset cases but with a milder disease course. We provide evidence that an alternative mechanism for disease, impairment of SAH rather than SAM transport, is the cause of adult onset *SLC25A26*-related mitochondrial disease.

## Results

### Patient 1 clinical summary

**Figure 1:**
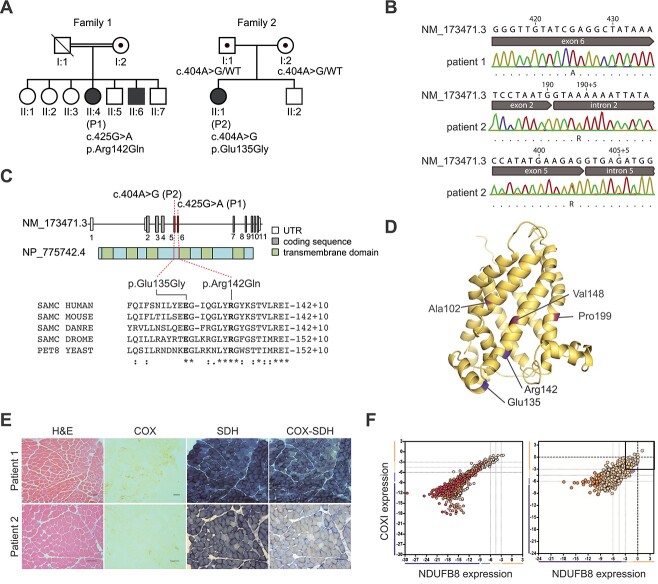
Genetic and biochemical characterization of patients with SLC25A26 variants. (**A**) Pedigree of individuals P1 and P2. (**B**) Sequencing chromatogram of genomic DNA aligned to *SLC25A26* (NM_173471.3). (**C**) Diagram representing the relative positions of the *SLC25A26* variants (NM_173471.3) and SLC25A26 alterations (GenBank: NP_775742.4). Amino acid alignments of five species show the regions of each variant. (**D**) Position of the variant amino acids in a modeled structure of human SLC25A26 (blue). Previously identified amino acids with disease association are marked in purple. (**E**) Standard histopathological analyses [Hematoxylin and eosin (H&E), cytochrome *c* oxidase (COX), succinate dehydrogenase (SDH) and sequential COX-SDH histochemistry] were undertaken in both patients, revealing a marked loss of COX activity and mitochondrial accumulation. Size bar = 100 μm. (**F**) Quadruple immunofluorescence analysis of NDUFB8 (complex I) and COXI (Complex IV) protein expression in patient muscle. Each dot represents the measurement from an individual muscle fiber, color co-ordinated according to its mitochondrial mass (low = blue, normal = beige, high = orange, very high = red). Gray dashed lines represent SD limits for classification of the fibers. Lines next to *x*- and *y*-axis represent the levels of NDUFB8 and COXI: beige = normal (> −3), light beige = intermediate positive (−3 to −4.5), light purple = intermediate negative (−4.5 to −6) and purple = deficient (< −6). Bold dashed lines represent the mean expression level of normal fibers. These data confirm the loss of NDUFB8 and COX1 expression in most fibers, consistent with the demonstration of multiple OXPHOS deficiencies.

A 29-year-old woman presented electively with a 2-year history of severe, progressive exercise intolerance, manifested by rapid fatigue, myalgia and cramps (Table 1). She reported that symptoms would arise after 2 min of walking (described as her legs ‘sticking’), necessitating rest in addition to rapidly increasing shortness of breath on exertion (SOBOE), palpitations and profound fatigue. Pain was the predominant symptom reported to limit mobility in addition to headache and nausea on more vigorous exertion. Features suggestive of a second wind phenomenon or myoglobinuria were not elicited. She also reported lower urinary symptoms in terms of frequency, urgency and incontinence. She had a history of mild eczema and vitamin D deficiency. Medications included cholecalciferol 800 Units/calcium carbonate 2.5 g one daily. Neurological examination revealed no PEO, ptosis or retinopathy. She had subtle neck flexor weakness (MRC 4+/5), proximal myopathy (MRC 4+/5) and hesitant tandem gait. Routine bloods including full blood count, random glucose, liver and renal profile, thyroid function, carnitine/acylcarnitine profile, urinary organic acids and creatine kinase (98 U/l) were normal. An uncuffed lactate sample, taken at rest, was 4.7 mmol/l (range 0.5–2.2). Urine was negative for ketones. A synacthen test, to monitor cortisol production, was performed and was normal. Nerve conduction studies and electromyography (EMG) showed intact sensory responses, intact motor responses with compact motor responses with no recurrent discharges. Concentric needle EMG of the right tibialis anterior showed a degree of motor unit remodeling with some polyphasic units with high frequency components, in keeping borderline myopathic changes. Cardiac evaluation, including an echocardiogram and ambulatory 24-h ECG recording, revealed normal left ventricular ejection fraction >55%, normal RV, no left ventricular hypertrophy, normal valves and sinus rhythm throughout with heart rates ranging between 61 and 128 bpm (mean HR 78 bpm) occasional VPBs and SVE were recorded (within normal limits and not of any prognostic significance). A cystometrogram confirmed detrusor overactivity and she was commenced on Solifenacin 5 mg daily. She was the fourth of seven children, born to consanguineous parents (second cousins) (Fig. 1A). A trial of dietary manipulation, Q10 and riboflavin supplements were of no benefit. A wheelchair for energy conservation purposes was prescribed. Her younger brother (sixth born) reported similar but milder symptoms of exercise intolerance. He has not been formally assessed clinically. Telephone consultation revealed he too had features, albeit milder of exercise intolerance. He reported being unable to run, increased SOBOE and fatigue on minimal exertion as well as rapidly increasing heart rate. He had modified his lifestyle due to his symptoms including employment (sedentary occupation).

**Table 1:** Clinical description

Patient	Case 1	Case 2
Sex	Female	Female
Ethnicity	Asian, Pakistani	White, European
Age at presentation	29 years	24 years
Variant	c.425G>A p.(Arg142Gln)	c.190+4A>G; c.404A>G p.(Glu135Gly)
Muscle histochemistry	Multiple fibers with internal nuclei; occasional necrotic fibers subsarcolemmal vacuoles++, excess glycogen	Predominance of type 1 fibers, increased lipid
	>95% COX-deficient fibers, occasional ragged red fibers	>90% COX-deficient fibers, occasional ragged red fibers
Muscle Respiratory chain enzyme activities	N/A	Complex I [0.009 (0.104–0.268)], Complex II + III [0.018 (0.040–0.204)], Complex IV [0.001 (0.014–0.034)]
Clinical features	History of exercise intolerance with muscle pain	History of exercise intolerance with muscle pain
	Diminished reflexes	Axial weakness
	Tachycardia (61–128 bpm)	Weight loss
	Low MCV 81.0 fl (83–101) Myopathic EMG; MRI: mild fatty infiltration of several, mainly posterior compartment thigh muscles	History of anemia
	Subtle proximal myopathy (adapted Gower’s maneuver positive)	Episodes of fever, abdominal pain and vomiting
	Hesitant tandem gait	Recurrent metabolic acidosis
Urinary organic acids	N/A	Increased 2-ketoglutarate & 2-hydroxyglutarate
Serum lactate	4.7 mmol/l (0.5–2.2)	Up to 8 mmol/l
CSF lactate	N/A	1.6 mmol/l
FGF21	N/A	4582 ng/l
CK	120 IU/l 30 nmol/l	600 IU/l N/A
PDH activity	0.47 nmol/mg protein/min (0.6–0.9 nmol/mg protein/min)	0.52 nmol/mg protein/min (0.6–0.9 nmol/mg protein/min)
CSF SAM	N/A	207 nmol/l (137–385)
CSF SAH	N/A	15 nmol/l (9–14)
Plasma SAM	N/A	93 nmol/l (65–130)
Plasma SAH	N/A	17 nmol/l (10–23)

N/A, not available or N/D, not determined.

### Patient 2 clinical summary

A 24-year-old female was admitted for emergency treatment with nausea, vomiting, abdominal pain, a temperature of 38.2°C and dehydration (Table 1). She had metabolic acidosis with pH 7.08, 9.6 mmol/l bicarbonate and lactate of 6 mmol/l. *Escherichia coli* was detected in urine and sepsis was suspected, although blood cultures were negative. Improvement was seen after administration of intravenous dextrose and co-amoxiclav. There were several further episodes presenting with a similar clinical picture and requiring hospital treatment. Mesenteric ischemia and bowel obstruction were suspected in view of persistent abdominal pain, vomiting, unexplained metabolic acidosis and high lactate, and an exploratory laparotomy was undertaken. No obstruction was detected; however, recurrent metabolic acidosis occurred postoperatively. Urine organic acid analysis detected increased lactate, ketones and 2-ketoglutarate. Improvement was seen with dextrose, bicarbonate and feeding, and she returned home coming back every few days for potassium, magnesium and iron replacement. There was no relevant family history (Fig. 1A); however, the patient had a personal history of poor exercise tolerance with muscle pain, plus a longstanding history of iron deficiency. There was no evidence of eye symptoms, diabetes, hearing problems or cardiac disease. She remains clinically stable with no further episodes of metabolic decompensation. She recently delivered a healthy male infant by caesarean section.

### Novel SLC25A26 variants identified by whole exome sequencing

Whole exome sequencing (Fig. 1B) revealed rare, damaging SLC25A26 (NM_173471.3) gene variants in both probands. Patient 1 was homozygous for a c.425G>A(p.Arg142Gln) substitution of a conserved amino acid residue, while Patient 2 had two heterozygous variants: c.190+4A>G and c.404A>G(p.Glu135Gly). Sanger sequencing of parental samples was used to establish heterozygosity and phase of the variants in Patient 2’s family, but additional familial samples were not available for Patient 1.

The c.425G>A(p.Arg142Gln) variant identified in Patient 1 has previously been detected as a heterozygous variant in 1 of 56 361 European individuals screened as part of the Genome Aggregation Project (gnomAD) but was not detected in other populations in this study (124 650 total individuals). Similarly, the c.190+4A>G and c.404A>G p.(Glu135Gly) variants in Patient 2 had been detected as heterozygous variants in 6 of 36 749 European individuals (1 of 49 425 individuals of other ethnic populations) and 1 of 38 952 European individuals (0 of 55 689 individuals of other ethnic populations), respectively.

*In silico* analysis predicted that the c.190+4A>G and c.404A>G variants of Patient 2 would lead to aberrant splicing in exons 3 and 5, respectively. Analysis of cDNA from fibroblasts from Patient 2 detected a shorter-than-expected transcript lacking exon 3, consistent with a splice defect due to the c.190+4A>G variant (Supplementary Material, Fig. S1A). This shorter transcript was also observed in control fibroblasts. Sequencing of cDNA showed complete skipping of exon 3 in patient fibroblasts not observed in control samples. The c.404A>G variant is predicted to form a cryptic splice donor site within exon 5, predicted to result in the deletion of the last 54 nucleotides of this exon and an 18 amino acid in-frame deletion. cDNA analysis identified only a small proportion of transcripts using the cryptic splice site in preference to the legitimate exon 5 donor site (Supplementary Material, Fig. S1B). Both missense variants affect residues which are highly conserved in eukaryotes (Fig. 1C), and *in silico* modeling places both variants to regions facing the mitochondrial matrix suggested to be important for domain structure and function (Fig. 1D). Of note, a predictive model of the murine structure with AlphaFold (34) suggests ionic interaction of Glu135 and Arg142 (Supplementary Material, Fig. S2).

### Patient muscle biopsies show mitochondrial biochemical defects

Histological and histochemical assessment of skeletal muscle sections from both patients showed evidence of mitochondrial dysfunction following sequential cytochrome *c* oxidase (COX)/succinate dehydrogenase histochemistry, demonstrating marked numbers of COX-deficient fibers (Fig. 1E). Quantitative quadruple IHC analyses confirmed extensive mitochondrial OXPHOS dysfunction, within most of the individual muscle fibers analyzed revealing complete loss of complex I and complex IV protein expression (Fig. 1F) (35). This was accompanied by a mild increase in mtDNA content in the skeletal muscle of both patients and evidence of variable mtDNA deletions in the muscle of Patient 2 (Supplementary Material, Fig. S1C), consistent with the patchy OXPHOS dysfunction. A diagnostic muscle biopsy from Patient 2 at 26 years of age revealed decreased activities of complexes I, II + III (succinate:cytochrome *c* reductase) and IV (Supplementary Material, Fig. S1D).

**Figure 2:**
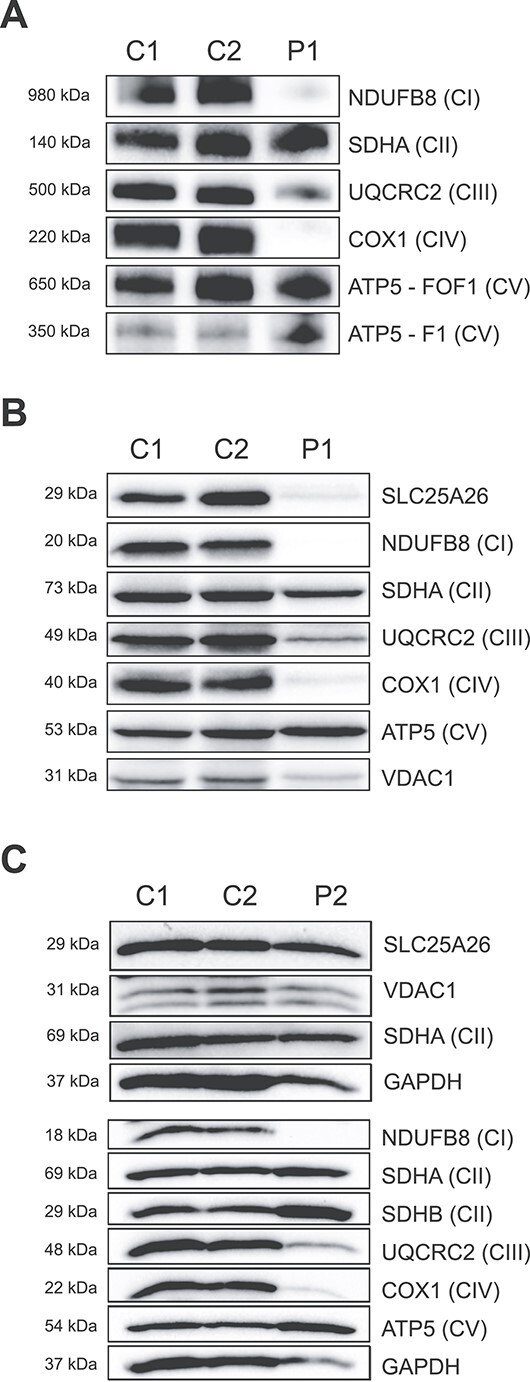
Protein expression analysis in patient muscle biopsies. (**A**) Blue-native polyacrylamide gel electrophoresis (BN-PAGE) of isolated skeletal muscle mitochondria (5.68 μg protein) from control and Patient 1, decorated with antibodies against the indicated subunits of NADH:ubiquinone oxidoreductase (CI), succinate:ubiquinone oxidoreductase (CII), ubiquinone:cytochrome *c* oxidoreductase (CIV) and ATP synthase (CV). (**B**, **C**) Sodium dodecyl sulfate (SDS)-PAGE of total protein skeletal muscle (39.98 μg protein) from control and Patient 1 (B) and Patient 2 (C), decorated with antibodies as in (A), as well as SLC25A26. VDAC1 or GAPDH was used as loading control.

Blue-native PAGE (BN-PAGE) analysis of muscle mitochondria from both patients showed defects in OXPHOS complex assembly, with decreased levels of assembled complexes I, III and IV, without affecting complexes II and V in Patient 1 (Fig. 2A), and a partially disassembled F_O_F_1_ subunit of ATP synthase in Patient 2 (Supplementary Material, Fig. S1E).

Denaturing SDS-PAGE further confirmed a loss of the complex I subunit NDUFB8 and the cytochrome *c* oxidase subunit COX1, as well as a milder decrease in the complex III subunit UQCRC2 (Fig. 2B and C). Furthermore, SLC25A26 levels were decreased in muscle from both patients, although to a lesser extent in Patient 2. Interestingly, BN-PAGE of fibroblasts from Patient 1 did not show abnormal OXPHOS complex formation or decreased respiratory chain enzyme activities (Supplementary Material, Fig. S3A and B).

### SAH transport across the mitochondrial membrane is impaired in a cell model

**Figure 3:**
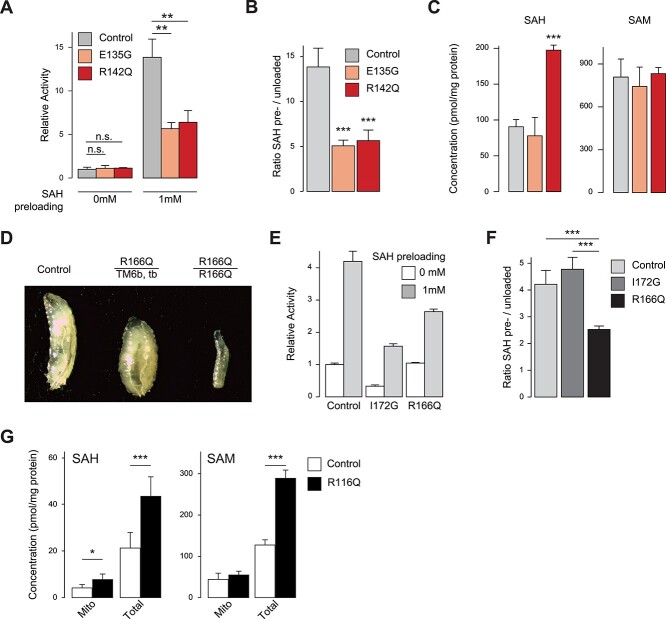
SAH carriage is impaired in MEF models. (**A**) Mitochondrial import of radiolabelled SAM with or without preincubation of mitochondria with SAH relative to Control (stable transfection of *slc25a26* KO MEFs with human *SLC25A26* cDNA) or E135G (*slc25a26* KO MEFs expressing the p.Glu135Gly variant) or R142Q (*slc25a26* KO MEFs expressing the p.Arg142Gln variant). (**B**) Same data as in (A) expressed as ratio. (**C**) Absolute levels of SAH and SAM in total cell metabolite extracts. (**D**) Larval phenotype at 4 days after egg laying (dae) of *wDah* control or larvae heterozygous for the p.Arg166Gln (R166Q) mutation balanced on *Tmb6, tubby* (tb), or homozygous for p.Arg166Gln (left to right). (**E**) Mitochondrial import of radiolabelled SAM with (grey bars) or without (white bars) preincubating mitochondria with 1 mm SAH relative to *wDah* controls or (**F**) as a ratio preincubated to untreated. I172G larvae were previously described (33) and express the Ile172Gly variant. (**G**) Absolute levels of SAH and SAM in fast mitochondrial enriched fractions or total larval metabolite extracts. ^*^P < 0.05, ^**^P < 0.01 and ^***^P < 0.001 with two-sided Student’s *t-*test (*n* = 3).

Fibroblasts from both patients presented with a mild decrease in PDH enzyme activity, consistent with the E2 subunit of PDH requiring mitochondrial localized SAM-dependent lipoic acid as a cofactor (Table 1). In order to functionally assess the consequences of p.Glu135Gly in the absence of the c.190+4A>G splice variant, we expressed the human c.404A>G(p.Glu135Gly) or c.425G>A(p.Arg142Gln) variants in previously established mouse embryonic fibroblasts (MEFs) deficient of *Slc25a26* (*Slc25a26* KO) (33). Overexpression of either patient variant was able to rescue the delayed growth and medium acidification of KO cells, recover lipoic acid levels (Supplementary Material, Fig. S4A) and normalize oxygen consumption rates to a similar degree as cells expressing human wild-type *SLC25A26* (Supplementary Material, Fig. S4B). Flux analysis of SAMC import capacity, using radiolabeled SAM did not reveal any defect (Fig. 3A). To test SAH export, we preincubated isolated mitochondria with unlabeled SAH to artificially increase intramitochondrial SAH levels prior to the addition of labeled SAM. While this amplified SAM uptake at the experimental endpoint in controls due to the antiporter action of SAMC, mitochondria from cells harboring either variant were indeed less sensitive to preincubation and failed to increase their import capacity to a similar extent, suggesting reduced SAH transport due to the p.Glu135Gly or p.Arg142Gln variants (Fig. 3A and B). Total cellular SAH levels were increased in cells overexpressing the p.Arg142Gln variant (Fig. 3C), compatible with observations made previously in *Dm* models (33). Interestingly, Patient 2, carrying the p.Glu135Gly variant, had marginally increased SAH levels in CSF (15 nmol/l, Table 1) (9–14). Additionally, cells expressing the p.Arg142Gly variant had increased levels of acetyl-CoA, FAD and NAD^+^ levels (Supplementary Material, Fig. S4C).

### p.Arg166Gln is larval lethal in *Dm*

Next, we generated a *Dm* model, using a previously established *Dm* knock-out line of *CG4743*, the *Dm* ortholog of *SLC25A26* (33). Using homologous recombination, we established a fly knock-in line expressing p.Arg166Gln (p.Arg142Gln in Patient 1) at endogenous levels (for details see Materials and Methods). *Dm* larvae homozygous for p.Arg166Gln were smaller at 4 days after egg lay compared to wild-type controls (Fig. 3D) and died as larvae before pupation. In agreement with the MEF models, no difference in SAM flux was observed in isolated mitochondria from mutant larvae (Fig. 3E). However, corroborating our observation in the cell models, pre-loading mitochondria with SAH again failed to increase SAM flux in mutant samples (Fig. 3E and F). This result is in contrast to those reported in a previously established patient-specific fly model carrying a p.Ile172Gly (p.Val199Gly in humans) substitution (33), which manifest a decreased SAM import capacity, but responded normally to SAH preloading (Fig. 3E and F). Targeted metabolite analysis in total or mitochondrial extracts showed a marked increase in SAH, but normal SAM levels in mitochondria from p.Arg166Gln larvae, while SAM levels were only increased in total extracts (Fig. 3G). Notably, total acetyl-CoA levels were also elevated, although the molecular mechanism is unclear (Supplementary Material, Fig. S5A). Collectively, these data point to a novel pathomechanism of two described SAMC variants with adult-onset mitochondrial disease, which is caused by defective SAH and not SAM carriage across the mitochondrial inner membrane. This is further supported by the observation that in contrast to previous results, we failed to rescue p.Arg166Gly larvae by supplementing their growth media with methionine or SAM, supporting an alternative and novel pathomechanism due to these *SAMC* variants (Supplementary Material, Fig. S5B).

**Figure 4:**
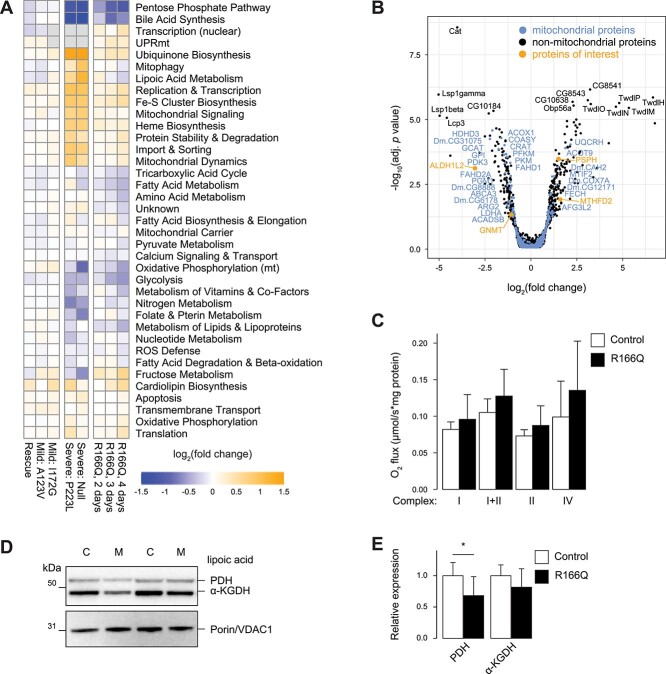
The *Dm* model expressing p.Arg166Gln is larval lethal. (**A**) Proteome landscape of previously established mutants at 4 dae (33) and *Arg166Gln* (R166Q) larvae at three timepoints after egg lay compared to *wDah* controls. I172G corresponds to larvae homozygous for a p.Ile172Gly mutation, P223L larvae are homozygous for p.Pro223Lys and null larvae are homozygous for non-expressed *slc25a26* alleles. Functional categories were published with MitoXplorer 2.0 (42) and are here vertically sorted according to their Euclidean distance. (*n* = 5 for previous models, *n* = 3 for each *Arg166Gln* group including matched *wDah* controls). (**B**) Proteome volcano plot of mutant against *wDah* control at 4 dae. Mitochondrial proteins as annotated in (42), proteins of interest are proposed markers of mitochondrial SAM deficient fly models (*n* = 3). (**C**) Mitochondrial oxygen consumption quantified on an OROBOROS oxygraphy respirometer (*n* = 4). (**D**) Representative lipoic acid levels on PDH and α-KGDH by denaturing SDS-PAGE and western blotting. C, Control; M, R166Q mutant. (**E**) Quantification of (D) (*n* = 6). ^*^P < 0.05, unpaired Student’s *t*-test.

Quantitative proteomics of mutant and control larvae at 2, 3 or 4 days after egg laying (dae) revealed a reduction in two annotated functional categories—pentose phosphate pathway and bile acid synthesis—while iron–sulfur cluster and ubiquinone biosynthesis proteins were upregulated (Fig. 4A, Supplementary Material, Table S1). This proteomic signature was comparable to previously described *SLC25A26 Dm* KO and p.Pro223Lys models (Fig. 4A) (33). Only two OXPHOS components were significantly altered, with UQCR-11 and COX7A proteins both showing increased expression (Fig. 4B). In agreement with this, oxygen consumption rates of mutant larvae were normal (Fig. 4C), with only a mild reduction of lipoylation on PDH and α-KGDH (Fig. 4D and E). However, we observed a loss of glycine-N-methyltransferase (GNMT) and accumulation of the serine biosynthesis enzyme phosphoserine phosphatase (PSPH) (Fig. 4B), which we previously reported to be biomarkers of mitochondrial methylation deficiency (33).

## Discussion

We present two adults with biallelic variants in the *SLC25A26* gene, encoding the mitochondrial SAM/SAH translocator, leading to mitochondrial disease caused primarily by deficient SAH export. Both patients presented with milder, albeit overlapping, phenotypes compared to the previously described neonatal cases (15). We therefore extend the clinical spectrum associated with inherited defects in this gene.

Members of the SLC25 carrier family consist of three homologous repeats of about 100 amino acids (36). Based on the structure of SLC25A4 (37), the three previously known *SLC25A26* missense variants affect residues located in the transmembrane region and are either directly involved in the transporting mechanism or residues in proximity to the pore. In contrast, the two novel variants c.404A>G p.(Glu135Gly) and c.425G>A p.(Arg142Gln) within repeat 2 are located further into the matrix and likely do not convey substrate specificity. Both residues are found in the second of three repeats of conserved signature motifs with the sequence 135-[DEL]GXXXX[YWF][RK]G-142. Glu135 is part of the E-R link II and contacts Arg115 of the E-R link I, which is the only described and highly conserved interaction required for stability of the domain structure (3). In this, the negative charge of glutamate is essential and the mutation to glycine will likely break this interaction. Variants affecting this link are pathogenic in other transporters (3). Arg142 is part of the cardiolipin binding site II that consists of the conserved motif [YWF][RK]G. Cardiolipins are tightly bound to SLC25 transporters and important for stability and function. The positively charged arginine is predicted to interact with the phosphate moiety of cardiolipin (38).

Several arguments point towards impaired SAH, and not SAM, transport efficiency caused by the two novel *SLC25A26* missense variants in these adult cases: first, the import of tritiated SAM into mitochondria was normal. Second, SAH dependency of SAM-import was decreased compared to controls. Third, we observed increased mitochondrial SAH levels while fourth, we saw no rescue of the larval developmental defect by methionine or SAM unlike in other mutants. We propose that the increase in mitochondrial SAH levels has a similar effect on mitochondrial function compared to mitochondrial SAM deficiency due to product inhibition of methyltransferases. This is supported by a comparable proteomic signature in Arg166Gln larvae compared to other models with mitochondrial SAM deficiency (33).

SAH in the cerebrospinal fluid (performed as an elective procedure to exclude central folate deficiency) was marginally increased in Patient 2 and this could be a result of impaired SAH transport across the mitochondrial membrane. A build-up of SAH inside mitochondria may mean that a proportion of cellular SAH is unable to be metabolized to homocysteine and adenosine by S-adenosylhomocysteine hydrolase, which takes place in the cytosol and therefore could contribute to elevated overall levels of SAH.

The functional studies for c.404A>G p.(Glu135Gly) and c.425G>A p.(Arg142Gln) provide evidence for altered SAH transport; however, the prosthetic lipoic acid group on PDH and α-KGDH are synthesized via a radical SAM reaction that does not yield SAH but an adenosyl radical and methionine. A global reduction in carrier levels can contribute to the molecular phenotype observed, and our results suggest that patients do present with reduced SLC25A26 protein levels in skeletal muscle samples. Whether the p.Arg142Gln variant in Patient 1 affects protein stability is unclear, but expression of the equivalent variant in *Dm* did not affect SAM import into mitochondria, suggesting the presence of at least some carrier protein that may explain the milder nature and later onset of disease. Patient 2 is compound heterozygous, where the c.190+4A>G variant leads to skipping of exon 3, which could result in reduced full-length protein expression. The combined impairment of SAM and SAH flux can therefore moderate reductions in all mitochondrial processes requiring SAM, such as lipoic acid synthesis and OXPHOS complex assembly. The contribution of the individual components of SAM flux and SAH clearance is unknown and might be important clinical markers to identify mitochondrial SAM deficiency. Given that the penetrance of splicing defects depends on genetic context and interactions with other genes products (39), the expression of this type of variant may explain the episodic course of Patient 2’s history. Indeed, the episodic course of the illness of Patient 2 with improved health between is a striking feature of the clinical history that influenced management. Provision of a lactate meter revealed outpatient values between 1.3 and 7.8, median 2.1 mm. This has enabled correlation of symptoms (‘restless painful legs’) with incipient biochemical decompensation and has thus far prevented further severe episodes.

We observed an increase in total acetyl-CoA levels, which were normal in fly mitochondria. The cytosolic enzyme citrate lyase (ACLY) converts citrate into acetyl-CoA and oxaloacetate and is responsible for the majority of nuclear acetyl-CoA used for epigenetic modifications on histones. It is possible that the cellular increase of SAM and SAH is due to high acetyl-CoA levels. The cytoplasmic enzyme GNMT converts SAM into sarcosine and is regulated by N-terminal acetylation, which increases the binding affinity to the allosteric inhibitor 5-methyltetrahydrofolate (40). In our p.Arg166Gln *Dm* model, GNMT is downregulated, suggesting reduced cytoplasmic SAM turnover in these flies at constant folate levels. Yet the mechanism underlying the observed increase in acetyl-CoA levels remains unclear.

Whereas the fly model presents a mutation-related phenotype, we failed to detect pathological features in the MEF models. This might be due to the artificial expression of human SAMC (‘overexpression’). Although the carriers themselves might be less effective per protein, an artificial large number of them could act in a compensatory manner. However, the MEF model serves to prove that the patient SAMC protein indeed has a SAH transporting defect and leaves SAM transport unaffected.

Our proteomic analyses did not suggest an activation of mTOR signaling, despite the 2.5-fold increase in total SAM levels in *Drosophila*. mTORC1 signaling is sensitive to cytosolic SAM but not SAH levels via SAMTOR (41), thus we would expect an induction of glycolysis and NADPH production in the pentose phosphate pathway in these models. Rather, it appears that the flow of metabolites through the TCA cycle is decreased, as suggested by downregulation of several key enzymes involved in amino acid metabolism and mild reduction of lipoic acid.

In summary, we present two adults with biallelic variants in the mitochondrial SAM/SAH carrier SLC25A26. In contrast to previously reported mitochondrial SAM deficiency causing childhood onset of a severe clinical pathology, we attribute the milder symptoms in both adult patients to failure in exporting the methylation by-product SAH from the mitochondrial matrix. Our findings highlight the potential of precision medicine to distinguish multiple molecular pathologies originating from variants in the same protein, which will be of importance in the future for appropriate clinical management and therapeutic decision-making.

## Materials and Methods

### Sequencing and variant calling

For patient 1, whole exome sequencing was performed by enrichment of coding regions with Nextera Rapid Exome Capture (Illumina, San Diego, US) and subsequent sequencing with 100 bp paired-end reads on an Illumina NextSeq500 platform. An in-house bioinformatics pipeline was applied to the raw FASTQ files. Briefly, PCR duplicates were removed using Fastuniq (41); Burrows-Wheeler Aligner (42) was used to align the resultant reads to the human reference genome (UCSC hg38); genetic variants were detected using Freebayes (43) and functionally annotated using Annovar (44). Variants were prioritized if exonic (coding) or in a splice-site region with a minor allele frequency of less than or equal to 0.01, according to external databases such as ExAC, 1000 genomes and gnomAD. The *in silico* prediction tools Polyphen 2 (http://genetics.bwh.harvard.edu/pph2/), CADD (45) and SIFT (http://sift.jcvi.org/) were used to assess pathogenicity of variants. Sanger sequencing was used to validate candidate variants. Genetic analysis in Patient 2 was performed using a targeted gene panel of 173 nuclear encoded mitochondrial disease genes. Library capture and enrichment were performed using the SureSelect^XT^ Clinical Research Exome v2 targeted enrichment system (Agilent Technologies, Santa Klara, US) and sequencing was performed on an Illumina HiSeq4000. Data was analyzed using an in-house pipeline. The *SLC25A26* sequence variants were confirmed by Sanger sequencing using Big Dye chemistry (Applied Biosystems, Waltham, US), capillary electrophoresis (3730 DNA Analyzer, Applied Biosystems) and analysis in SeqScanner v1.0 (Applied Biosystems) and Mutation Surveyor software (v5.1.2). Splice sites were predicted *in silico* with SpliceSiteFinder-like, NNSplice and MaxEntScan accessed through Alamut Visual v2.11 (Interactive Biosoftware, Rouen, France).

### RNA analysis

RNA was purified from patient fibroblasts (QIAamp RNA Blood Mini Kit, Qiagen, Hilden, Germany) and reverse transcription using random primers was performed to create a cDNA library (SuperScript IV First-Strand Synthesis System, Invitrogen, Waltham, US). Analysis of *SLC25A26* transcripts was performed by PCR of cDNA using specific oligonucleotides designed to amplify exons 2–4 and exons 3–6. Products were analyzed by agarose gel electrophoresis and Sanger sequencing (described above).

### Antibodies

Lipoic acid (437695, Calbiochem/Merck, Darmstadt, Germany); Hsp60 (AB1-SPA-807-E, Enzo Lifesciences Farmingdale, US); Human OXPHOS cocktail (ab110411, Abcam, Cambridge, UK; 1:1000); VDAC1 (ab14734, Abcam; 1:5000); SLC25A26 (ab175209, Abcam; 1:1000); GAPDH (600004, Proteintech; 1:5000) SDHA (ab14715, Abcam; 1:1000), COXI (ab14705, Abcam; 1:1000); NDUFB8 (ab110242, Abcam; 1:1000); UQCRC2 (ab14745, Abcam; 1:1000); ATP5A (ab14748, Abcam; 1:1000). Secondary antibodies used include Polyclonal Donkey Anti-Rabbit Ig/HRP (NA9340V, SigmaAldrich, St. Louis, US); Polyclonal Sheep Anti-Mouse Ig/HRP (NA9310V, SigmaAldrich); Polyclonal Rabbit Anti-Mouse Ig/HRP (P0161, Dako/Agilent; 1:2000) and Polyclonal Swine Anti-Rabbit Ig/HRP (P0399, Dako; 1:3000).

### Chemicals

S-(5′-Adenosyl)-l-methionine chloride dihydrochloride (A7007), crystalline S-(5′-Adenosyl)-l-homocysteine (A9384), Nicotinamide Adenine Dinucleotide Phosphate (N5755), Acetyl-Coenzyme A (A2056), Coenzyme A (C4812) and Flavine Adenine Dinucleotide (F6625) were obtained from Sigma-Aldrich. Nicotinamide Adenine Dinucleotide (10127964001) was from Roche Basel, Switzerland. The metabolomics standard SAMd3 (D-4093) was bought from CDN Isotopes Pointe-Claire, Canada, SAHd4 was from Cayman Chemical (9000372); tritiated SAM-[methyl-2H] at 250 μCi (NET155V250UC) was from PerkinElmer Waltham, US. Pierce™ Trypsin, MS-grade (90059) was from ThermoFisher Scientific Waltham, US.

### Plasmids

pGE-attB-GMR (GenBank FH791037), pBSSVD2005 (Addgene, Watertown, US 21826).

### Fly lines

*Dm* genetic background was Wolbachia-free white Dahomey (*wDah*). Knock-in flies were generated in the background of a *CG4743* knockout line (RRID:BDSC_92361) (32) with the genotype*;;CG4743^n1^*[*attP,w+*]/*TM6b,tb,GFP*. The *w+* marker was first removed by crossings to a Cre-expressing fly line (BL#766). *CG4743^c.496_497delinsCA^* encoding Arg166Gln was sequencing-verified and cloned into pGE-GMR-attB containing introns and 150 bp 5′-UTR. The plasmid was injected together with PhiC31 into *CG4743* knockout embryos (BestGene Inc. Chino Hills, US), and red-eyed transformants were backcrossed into a Wolbachia-free white Dahomey (*wDah*) background for six generations.

### Fly husbandry

Flies were bred on a standard yeast-sugar-agar medium (46). Lines were kept and experiments performed at 25°C, 60% humidity and a 12 h:12 h light:dark cycle. For experimental crosses, mutant and control flies were put to lay for 8 h and grown for 4 days. Larvae were then picked in ddH2O for subsequent experiments. The genetic background *wDah* line was used as a control. Parental lines for homozygous *CG4743* mutants were kept as a balanced heterozygous cross over *TM6b, tb, GFP*.

### Generation and cell culture of MEF lines

*Slc25a26* KO MEFs were previously generated (32). Human *SLC25A26* sequences were obtained for control, c.404A>G (p.Glu135Gly) and c.425G>A (p.Arg142Gln) from (GenScript, Piscataway Township, US, custom) and cloned into the retroviral pBABE-puromycin vector with Gateway technology (ThermoFisher Scientific), followed by transfection into Phoenix Amphotropic cells using Lipofectamine 3000 (Thermo Fisher Scientific). Retrovirus containing medium was filtered (0.45 μm; Sarstedt, Nümbrecht, Germany) and added to immortalized *Slc25a26* KO MEFs after the addition of polybrene (4 μg/ml). Stably transduced cells were cultured with DMEM high glucose, GlutaMAX (Thermo Fisher Scientific) supplemented with 10% fetal bovine serum (Thermo Fisher Scientific), 1% penicillin/streptomycin (Thermo Fisher Scientific), uridine (50 μg/ml; Sigma-Aldrich) and maintained under puromycin selection (1.5 μg/ml).

### Bisulfite pyro-sequencing

One μg of RNA was bisulfite converted with the EZ RNA Methylation Kit (Zymo Research Irvine, US; R5001), and reverse transcribed with the QIAGEN PyroMark RT kit 978801, using an optimized pair of one biotinylated and unmodified primer. Ten microliters of cDNA were enriched with Streptavidin-coupled sepharose beads, and pyro-sequencing was performed on a PyroMark Q24 system (QIAGEN; 9001514) according to manufacturer’s instructions.

### Mitochondrial preparations

Fly mitochondria for SAM import assay and Western blotting were prepared as previously described (46). For SAM import, MEF mitochondria were isolated following the procedure described in ref. (47). For metabolite quantification, fly mitochondria were isolated in 1 ml KBPS (136 mm KCl, 10 mm KH_2_PO_4_, pH 7.25). Cell detritus was removed by spinning at 1000*g*, and mitochondria in the supernatant were pelleted at 3000*g* and washed once. Mitochondrial mass was estimated by a Qubit protein quantification assay (ThermoFisher Scientific).

### SAM mitochondrial import assay

Mitochondria were isolated as described above. Pellets of 50 and 15 μg from fly and mouse, respectively, were resuspended in 98 μl import assay buffer (100 mm mannitol, 10 mm sodium succinate, 80 mm KCl, 5 mm MgCl_2_, 1 mm KH2PO4, 25 mm HEPES, 5 mm ATP, 200 nm GTP, 6 mm creatine phosphate, 60 ng/ml creatine kinase and 60 mg/l of each amino acid Ala, Arg, Asp, Asn, Cys, Glu, Gln, Gly, His, Ile, Leu, Lys, Met, Phe, Pro, Ser, Thr, Trp, Tyr, Val, pH 7.4). SAH preloading was achieved by incubating mitochondria in 100 μl SAH-saturated import assay buffer on ice for 30 min, followed by pelleting at 9000*g* for 2 min and resuspension of the pellet in 98 μl assay buffer without SAH. Two microliters of radiolabelled SAM-[methyl-^3^H] (PerkinElmer) were added, and incubated at 30°C for 20 and 5 min in fly and mouse mitochondria, respectively. Mitochondria were pelleted at 9000*g*, 2 min, 4°C and washed twice in 200 μl assay buffer supplemented with 1 mm unlabeled SAM. Mitochondria were frozen, resuspended in 50 μl KPBS and quantified in 3 ml UltimaGold scintillation cocktail (PerkinElmer) and mean activity was acquired over 1 min with a liquid scintillation counter (LKB Instruments, Mount Waverly, Australia).

### Steady-state levels of metabolites

Metabolite extraction from mitochondrial pellets or total homogenates was done with ice-cold 80% methanol spiked with labeled internal standards SAMd3 and SAHd4 incubated on ice for 15 min. Supernatants were collected after centrifugation (15 000*g*, 15 min, 4°C) and evaporated to dryness in a Concentrator Plus (Eppendorf, Hamburg, Germany). Dried extracts were resuspended in mobile phase A. Instrument (Waters XEVO TQ-MS, Milford, US) was run in multiple reaction monitoring (MRM) in positive mode (ES+) with mass transitions detailed in ref. (32). ESI capillary voltage was 3.0 kV, extractor voltage 3 V, RF lens voltage 0.1 V, desolvation gas (hydrogen) flow 1200 l/h at 450°C and cone gas (argon) flow was 150 l/h with source block at 150°C. Separation was achieved in an Acquity™ UPLC© BEH C18 1.7 μm, 2.1 × 100 mm column (Waters 186002352) at 60°C. Partial loop injection with needle overfill with a volume of 10 μl was performed. A linear gradient of mobile phase A (10 mm ammonium formate, pH 5.7) and phase B (100% acetonitrile) was used. Flow was kept constant at 0.6 ml/min (100% A) for 1 min, followed by an increase of B to 100% in 2.5 min and kept for 0.6 min. Phase A was set back to 100% in 0.1 min and kept for 2 min. Calibration curves (0–1000 pmol) were based on determination of analyte/IS peak area ratios versus analyte amount. Data acquisition and peak integration were all performed with Masslynx 4.1 software (Waters).

### SDS gel electrophoresis and Western blotting

Mitochondrial pellets were lysed in RIPA buffer, incubated on ice for 15 min and cell detritus was removed at 12 000*g* for 15 min and 4°C. The supernatant was mixed with NuPAGE™ LDS sample buffer and reducing reagent according to the manufacturer’s instructions (ThermoFisher Scientific) and an equivalent of 15 μg mitochondrial protein was loaded on 4–12% NuPAGE™ Bis-Tris, 1.0 mm gel. Gels were run for 50 min at 200 V in MOPS buffer and transferred with an iBlot™ system (Thermo Fisher Scientific) onto PVDF membranes. These were then blocked in 5% milk in Tris buffer saline with 1% Tween 20. Immunoblotting was performed using standard techniques and developed with Clarity Western ECL substrate (Bio-Rad Laboratories, Hercules, US).

### Peptide sample preparation and mass spectrometry

Larval peptides were prepared as previously described (46). Two micrograms of samples were injected onto an UltiMate 3000 nano UPLC equipped and chromatographic separation was achieved with a 50-cm-long C18 EASY-Spray column (Thermo Fisher Scientific) at 55°C. The following gradient was applied: 4–26% of solvent B (98% acetonitrile and 0.1% FA) in 90 min, 26–95% of solvent B in 5 min and 95% of solvent B for 5 min at a flow rate of 300 nl/min. The mass spectrometric acquisition on a Q Exactive HF Hybrid Quadrupole-Orbitrap mass spectrometer (Thermo Fisher Scientific, Bremen, Germany) comprised one survey full mass spectrum ranging from a mass/charge ratio (*m*/*z*) of 350–1600 at a resolution of *R* = 120 000 (at *m*/*z* 200) targeting 5 × 106 ions for a maximum injection time of 100 ms, followed by data-dependent higher-energy collision dissociation fragmentations of maximum 18 most intense precursor ions with a charge state 2+ to 7+, using 45-s dynamic exclusion. The tandem mass spectra were acquired with a resolution of *R* = 60 000, targeting 2 × 105 ions for a maximum injection time of 54 ms, setting isolation width to m/z 1.4 and normalized collision energy to 33% setting first mass at *m*/*z* 100.

### Proteomic data analysis

Spectra were search with Quandenser v0.061 (48) against a *Dm* UniProt library (August 2020) at standard settings separately for each age comparison. Result sheets were processed with R v4.0.3 in RStudio 1.2.5001.

### Respirometry

Mitochondrial respiration was assessed in dissected larvae or permeabilized MEFs cells at 25 or 37°C, respectively, using an Oxygraph-2K respirometer (Oroboros Instruments, Innsbruck, Austria). Samples were resuspended in respiratory buffer (0.5 mm EGTA, 3 mm MgCl_2_.6 H_2_O, 60 mm lactobionate, 20 mm taurine, 10 mm KH_2_PO_4_, 20 mm HEPES, 110 mm sucrose and 1 mg/ml BSA, pH 7.1) and permeabilized with digitonin. Complex I-dependent respiration was assessed by the addition of pyruvate (5 mm), glutamate (10 mm) and malate (2 mm), followed by the addition of ADP (2.5 mm). Succinate (10 mm) was added to determine complex I + II-dependent respiration, and then rotenone (0.5 μm) was added to determine complex II-dependent respiration. Non-mitochondrial respiration was determined by the addition of antimycin A (2.5 μm). Finally, complex IV activity was assessed by the addition of TMPD (0.5 mm) and ascorbate (2 mm), followed by the addition of potassium cyanide (1 mm). Oxygen consumption rate was normalized to protein content measured by the BCA assay.

### Structural modeling

Human SLC25A26 was modeled based on SLC25A4 (PDB: 1OKC (35)) with Modeller v9.21 (49). Structures were visualized with PyMOL 2.3.4.

## Data availability

The mass spectrometry proteomics data have been deposited to the ProteomeXchange Consortium via the PRIDE partner repository (50) with the dataset identifiers PXD026900. The generated CG4743 knock-in fly lines are available through the Bloomington Drosophila Stock Center at the Indiana University (RRID:BDSC_92367).

## Supplementary Material

Schober_et_al_Supplemental_figures_ddac002

Schober_et_al_Table_S1_ddac002

## References

[1] Ruprecht, J.J. and Kunji, E.R.S. (2019) The SLC25 mitochondrial carrier family: structure and mechanism. Trends Biochem. Sci., 45, 244–258. 31787485 10.1016/j.tibs.2019.11.001 PMC7611774

[2] Palmieri, F. (2013) The mitochondrial transporter family SLC25: Identification, properties and physiopathology. Mol. Asp. Med., 34, 465–484. 10.1016/j.mam.2012.05.005 23266187

[3] Kunji, E.R.S., King, M.S., Ruprecht, J.J. and Thangaratnarajah, C. (2020) The SLC25 carrier family: important transport proteins in mitochondrial physiology and pathology. Physiology, 35, 302–327. 32783608 10.1152/physiol.00009.2020 PMC7611780

[4] Palmieri, F., Scarcia, P. and Monné, M. (2020) Diseases caused by mutations in mitochondrial carrier genes SLC25: a review. Biomol. Ther., 10, 655. 10.3390/biom10040655 PMC7226361 32340404

[5] Kaukonen, J., Juselius, J.K., Tiranti, V., Kyttälä, A., Zeviani, M., Comi, G.P., Keränen, S., Peltonen, L. and Suomalainen, A. (2000) Role of adenine nucleotide translocator 1 in mtDNA maintenance. Science, 289, 782–785. 10926541 10.1126/science.289.5480.782

[6] Fontanesi, F., Palmieri, L., Scarcia, P., Lodi, T., Donnini, C., Limongelli, A., Tiranti, V., Zeviani, M., Ferrero, I. and Viola, A.M. (2004) Mutations in AAC2, equivalent to human adPEO-associated ANT1 mutations, lead to defective oxidative phosphorylation in *Saccharomyces cerevisiae* and affect mitochondrial DNA stability. Hum. Mol. Genet., 13, 923–934. 15016764 10.1093/hmg/ddh108

[7] Palmieri, L., Alberio, S., Pisano, I., Lodi, T., Meznaric-Petrusa, M., Zidar, J., Santoro, A., Scarcia, P., Fontanesi, F., Lamantea, E. et al. (2005) Complete loss-of-function of the heart/muscle-specific adenine nucleotide translocator is associated with mitochondrial myopathy and cardiomyopathy. Hum. Mol. Genet., 14, 3079–3088. 16155110 10.1093/hmg/ddi341

[8] Thompson, K., Majd, H., Dallabona, C., Reinson, K., King, M.S., Alston, C.L., He, L., Lodi, T., Jones, S.A., Fattal-Valevski, A. et al. (2016) Recurrent de novo dominant mutations in SLC25A4 cause severe early-onset mitochondrial disease and loss of mitochondrial DNA copy number. Am. J. Hum. Genet., 99, 860–876. 27693233 10.1016/j.ajhg.2016.08.014 PMC5065686

[9] King, M.S., Thompson, K., Hopton, S., He, L., Kunji, E.R.S., Taylor, R.W. and Ortiz-Gonzalez, X.R. (2018) Expanding the phenotype of de novo SLC25A4-linked mitochondrial disease to include mild myopathy. Neurology Genetics, 4, e256. 30046662 10.1212/NXG.0000000000000256 PMC6055355

[10] Wibom, R., Lasorsa, F.M., Töhönen, V., Barbaro, M., Sterky, F.H., Kucinski, T., Naess, K., Jonsson, M., Pierri, C.L., Palmieri, F. et al. (2009) AGC1 deficiency associated with global cerebral hypomyelination. New Engl. J. Med., 361, 489–495. 19641205 10.1056/NEJMoa0900591

[11] Falk, M.J., Li, D., Gai, X., McCormick, E., Place, E., Lasorsa, F.M., Otieno, F.G., Hou, C., Kim, C.E., Abdel-Magid, N. et al. (2014) AGC1 deficiency causes infantile epilepsy, abnormal myelination, and reduced N-acetylaspartate. JIMD Reports, 14, 77–85. 24515575 10.1007/8904_2013_287 PMC4213337

[12] Kobayashi, K., Sinasac, D.S., Iijima, M., Boright, A.P., Begum, L., Lee, J.R., Yasuda, T., Ikeda, S., Hirano, R., Terazono, H. et al. (1999) The gene mutated in adult-onset type II citrullinaemia encodes a putative mitochondrial carrier protein. Nat. Genet., 22, 159–163. 10369257 10.1038/9667

[13] Okano, Y., Ohura, T., Sakamoto, O. and Inui, A. (2019) Current treatment for citrin deficiency during NICCD and adaptation/compensation stages: strategy to prevent CTLN2. Mol. Genet. Metab., 127, 175–183. 31255436 10.1016/j.ymgme.2019.06.004

[14] Agrimi, G., Noia, M.A.D., Marobbio, C.M.T., Fiermonte, G., Lasorsa, F.M. and Palmieri, F. (2004) Identification of the human mitochondrial S-adenosylmethionine transporter: bacterial expression, reconstitution, functional characterization and tissue distribution. Biochem. J., 379, 183–190. 14674884 10.1042/BJ20031664 PMC1224042

[15] Kishita, Y., Pajak, A., Bolar, N.A., Marobbio, C.M.T., Maffezzini, C., Miniero, D.V., Monné, M., Kohda, M., Stranneheim, H., Murayama, K. et al. (2015) Intra-mitochondrial methylation deficiency due to mutations in SLC25A26. Am. J. Hum. Genet., 97, 761–768. 26522469 10.1016/j.ajhg.2015.09.013 PMC4667130

[16] Ducker, G.S. and Rabinowitz, J.D. (2017) one-carbon metabolism in health and disease. Cell Metab., 25, 27–42. 27641100 10.1016/j.cmet.2016.08.009 PMC5353360

[17] Fontecave, M., Atta, M. and Mulliez, E. (2004) S-adenosylmethionine: nothing goes to waste. Trends Biochem. Sci., 29, 243–249. 15130560 10.1016/j.tibs.2004.03.007

[18] Petrossian, T.C. and Clarke, S.G. (2011) Uncovering the human methyltransferasome*. Mol. Cell. Proteomics, 10(M110), 000976. 10.1074/mcp.M110.000976 PMC3013446 20930037

[19] Clarke, S.G. (2013) Protein methylation at the surface and buried deep: thinking outside the histone box. Trends Biochem. Sci., 38, 243–252. 23490039 10.1016/j.tibs.2013.02.004 PMC3634909

[20] Metodiev, M.D., Spåhr, H., Polosa, P.L., Meharg, C., Becker, C., Altmueller, J., Habermann, B., Larsson, N.-G. and Ruzzenente, B. (2014) NSUN4 is a dual function mitochondrial protein required for both methylation of 12S rRNA and coordination of mitoribosomal assembly. PLoS Genet., 10, e1004110. 24516400 10.1371/journal.pgen.1004110 PMC3916286

[21] Voigts-Hoffmann, F., Hengesbach, M., Kobitski, A.Y., Aerschot, A. van, Herdewijn, P., Nienhaus, G.U. and Helm, M. (2007) A methyl group controls conformational equilibrium in human mitochondrial tRNA Lys. J. Am. Chem. Soc., 129, 13382–13383. 17941640 10.1021/ja075520+

[22] Vilardo, E., Nachbagauer, C., Buzet, A., Taschner, A., Holzmann, J. and Rossmanith, W. (2012) A subcomplex of human mitochondrial RNase P is a bifunctional methyltransferase—extensive moonlighting in mitochondrial tRNA biogenesis. Nucleic Acids Res., 40, 11583–11593. 23042678 10.1093/nar/gks910 PMC3526285

[23] Suzuki, T., Yashiro, Y., Kikuchi, I., Ishigami, Y., Saito, H., Matsuzawa, I., Okada, S., Mito, M., Iwasaki, S., Ma, D. et al. (2020) Complete chemical structures of human mitochondrial tRNAs. Nat. Commun., 11, 4269. 32859890 10.1038/s41467-020-18068-6 PMC7455718

[24] Suzuki, T. and Suzuki, T. (2014) A complete landscape of post-transcriptional modifications in mammalian mitochondrial tRNAs. Nucleic Acids Res., 42, 7346–7357. 24831542 10.1093/nar/gku390 PMC4066797

[25] Spåhr, H., Habermann, B., Gustafsson, C.M., Larsson, N.-G. and Hallberg, B.M. (2012) Structure of the human MTERF4–NSUN4 protein complex that regulates mitochondrial ribosome biogenesis. Proc. Natl. Acad. Sci., 109, 15253–15258. 22949673 10.1073/pnas.1210688109 PMC3458362

[26] Rhein, V.F., Carroll, J., Ding, S., Fearnley, I.M. and Walker, J.E. (2013) NDUFAF7 methylates Arginine 85 in the NDUFS2 subunit of human complex I. J. Biol. Chem., 288, 33016–33026. 24089531 10.1074/jbc.M113.518803 PMC3829151

[27] Rhein, V.F., Carroll, J., He, J., Ding, S., Fearnley, I.M. and Walker, J.E. (2014) Human METTL20 methylates lysine residues adjacent to the recognition loop of the electron transfer flavoprotein in mitochondria. J. Biol. Chem., 289, 24640–24651. 25023281 10.1074/jbc.M114.580464 PMC4148887

[28] Rhein, V.F., Carroll, J., Ding, S., Fearnley, I.M. and Walker, J.E. (2017) Human METTL12 is a mitochondrial methyltransferase that modifies citrate synthase. FEBS Lett., 591, 1641–1652. 28391595 10.1002/1873-3468.12649 PMC5518231

[29] Małecki, J.M., Willemen, H.L.D.M., Pinto, R., Ho, A.Y.Y., Moen, A., Eijkelkamp, N. and Falnes, P.Ø. (2019) Human FAM173A is a mitochondrial lysine-specific methyltransferase that targets adenine nucleotide translocase and affects mitochondrial respiration. J. Biol. Chem., 294, 11654–11664. 31213526 10.1074/jbc.RA119.009045 PMC6682728

[30] Barkovich, R.J., Shtanko, A., Shepherd, J.A., Lee, P.T., Myles, D.C., Tzagoloff, A. and Clarke, C.F. (1997) Characterization of the COQ5 gene from *Saccharomyces cerevisiae* evidence for a C-methyltransferase in ubiquinone biosynthesis. J. Biol. Chem., 272, 9182–9188. 9083049 10.1074/jbc.272.14.9182

[31] Clarke, C.F., Williams, W. and Teruya, J.H. (1991) Ubiquinone biosynthesis in *Saccharomyces cerevisiae*. Isolation and sequence of COQ3, the 3,4-dihydroxy-5-hexaprenylbenzoate methyltransferase gene. J. Biol. Chem., 266, 16636–16644. 1885593

[32] Miller, J.R., Busby, R.W., Jordan, S.W., Cheek, J., Henshaw, T.F., Ashley, G.W., Broderick, J.B., Cronan, J.E. and Marletta, M.A. (2000) *Escherichia coli* LipA is a lipoyl synthase: in vitro biosynthesis of lipoylated pyruvate dehydrogenase complex from octanoyl-acyl carrier protein. Biochemistry, 39, 15166–15178. 11106496 10.1021/bi002060n

[33] Schober, F.A., Moore, D., Atanassov, I., Moedas, M.F., Clemente, P., Végvári, Á., Fissi, N.E., Filograna, R., Bucher, A.-L., Hinze, Y. et al. (2021) The one-carbon pool controls mitochondrial energy metabolism via complex I and iron-sulfur clusters. Sci. Adv., 7, eabf0717. 33608280 10.1126/sciadv.abf0717 PMC7895438

[34] Jumper, J., Evans, R., Pritzel, A., Green, T., Figurnov, M., Ronneberger, O., Tunyasuvunakool, K., Bates, R., Žídek, A., Potapenko, A. et al. (2021) Highly accurate protein structure prediction with AlphaFold. Nature, 596, 583–589. 34265844 10.1038/s41586-021-03819-2 PMC8371605

[35] Rocha, M.C., Grady, J.P., Grünewald, A., Vincent, A., Dobson, P.F., Taylor, R.W., Turnbull, D.M. and Rygiel, K.A. (2015) A novel immunofluorescent assay to investigate oxidative phosphorylation deficiency in mitochondrial myopathy: understanding mechanisms and improving diagnosis. Sci. Rep., 5, 15037. 26469001 10.1038/srep15037 PMC4606788

[36] Saraste, M. and Walker, J.E. (1982) Internal sequence repeats and the path of polypeptide in mitochondrial ADP/ATP translocase. FEBS Lett., 144, 250–254. 6288471 10.1016/0014-5793(82)80648-0

[37] Pebay-Peyroula, E., Dahout-Gonzalez, C., Kahn, R., Trézéguet, V., Lauquin, G.J.-M. and Brandolin, G. (2003) Structure of mitochondrial ADP/ATP carrier in complex with carboxyatractyloside. Nature, 426, 39–44. 14603310 10.1038/nature02056

[38] Duncan, A.L., Ruprecht, J.J., Kunji, E.R.S. and Robinson, A.J. (2018) Cardiolipin dynamics and binding to conserved residues in the mitochondrial ADP/ATP carrier. Biochim. Biophys. Acta Biomembr., 1860, 1035–1045. 29366674 10.1016/j.bbamem.2018.01.017 PMC5988563

[39] Baralle, D., Lucassen, A. and Buratti, E. (2009) Missed threads. EMBO Rep., 10, 810–816. 19648957 10.1038/embor.2009.170 PMC2726684

[40] Luka, Z., Loukachevitch, L.V. and Wagner, C. (2008) Acetylation of N-terminal valine of glycine N-methyltransferase affects enzyme inhibition by folate. Biochim. Biophys. Acta Proteins Proteom., 1784, 1342–1346. 10.1016/j.bbapap.2008.04.016 PMC2576408 18501206

[41] Gu, X., Orozco, J.M., Saxton, R.A., Condon, K.J., Liu, G.Y., Krawczyk, P.A., Scaria, S.M., Harper, J.W., Gygi, S.P. and Sabatini, D.M. (2017) SAMTOR is an S-adenosylmethionine sensor for the mTORC1 pathway. Science, 358, 813–818. 29123071 10.1126/science.aao3265 PMC5747364

[42] Yim, A., Koti, P., Bonnard, A., Marchiano, F., Dürrbaum, M., Garcia-Perez, C., Villaveces, J., Gamal, S., Cardone, G., Perocchi, F. et al. (2019) mitoXplorer, a visual data mining platform to systematically analyze and visualize mitochondrial expression dynamics and mutations. Nucleic Acids Res., 48, 605–632. 10.1093/nar/gkz1128 PMC6954439 31799603

