# Supplementary material for: Pathogenic SLC25A26 variants impair SAH transport activity causing mitochondrial disease
Source: Hum Mol Genet. 2022 Jan 13;31(12):2049–62. doi: 10.1093/hmg/ddac002 (PMC9239748; doi:10.1093/hmg/ddac002)
Supplement: Schober_et_al_Supplemental_figures_ddac002 [file schober_et_al_supplemental_figures_ddac002.zip › Schober_et_al_Supplemental_figures_ddac002.pdf]

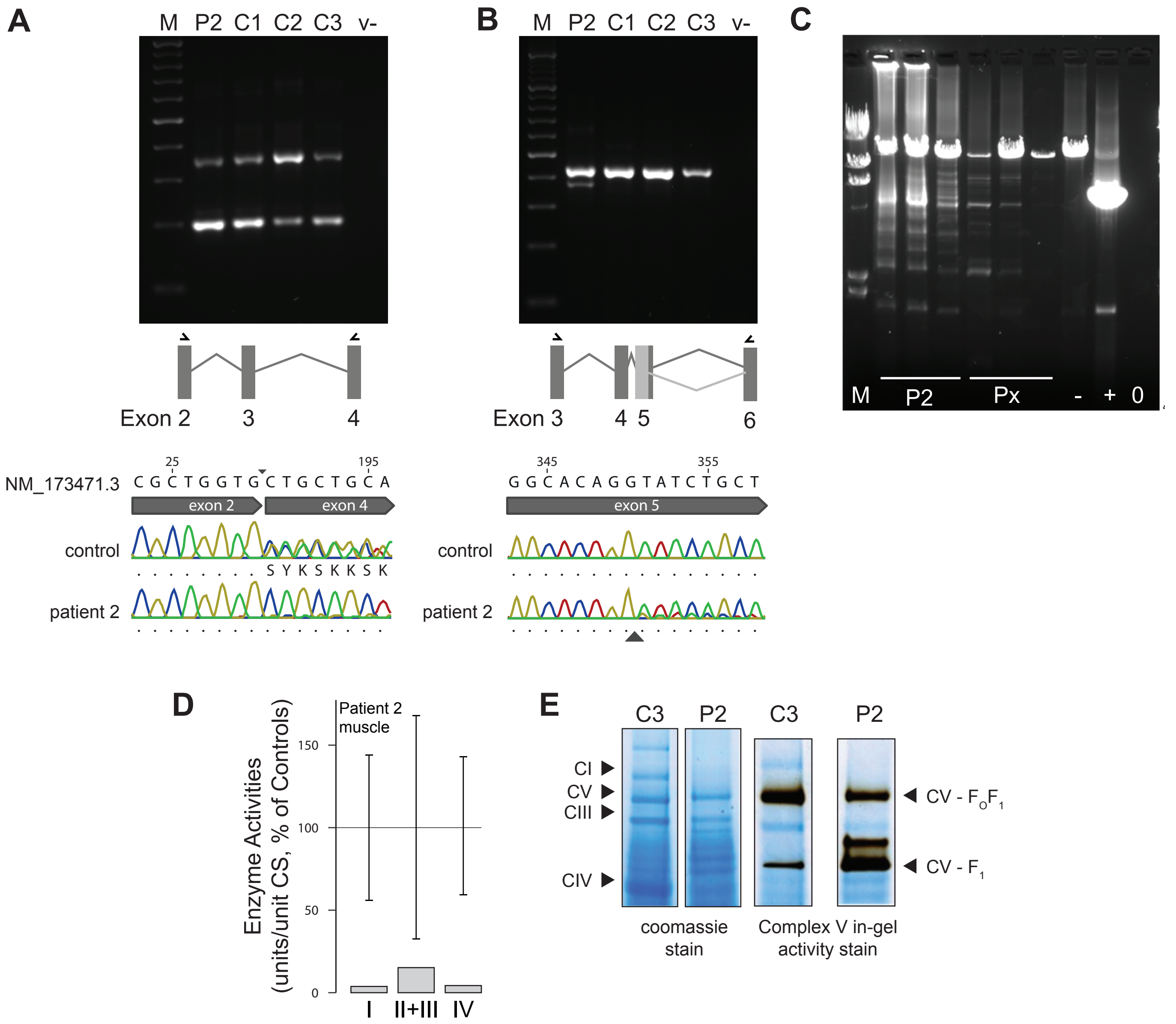

Fig. S1. Characterisation of Patient 2. (**A, B**) Agarose gel electrophoresis after PCR over the indicated genomic regions. M: marker; P2: patient 2; C1-3: control 1 to 3; v-: negative PCR control. Chromatograms of sequence analysis of the respective PCRs is shown below. Arrows indicate splice sites. (**B**) Use of an alternative splice donor site within exon 5 (black triangle) creates a 54 nucleotide deletion from the 3′ end of exon 5 in a small proportion of transcripts in Patient 2. (**C**) Long range PCR amplicons of 12.7 kb of the mitochondrial genome in muscle DNA from patient 2 (P2, lanes 2-4 at three different template dilutions). M, Marker; Px, unrelated patients; -, healthy control; +, single mtDNA rearrangement control; 0, no template control. (**D**) Isolated respiratory chain enzyme activities for NADH:ubiquinone oxidoreductase (I), succinate:cytochrome c oxidoreductase (SCR; II+III) and cytochrome c oxidase (IV). Normal intervals as indicated. (**E**) Coomassie stain (left panel) of a BN-PAGE of isolated skeletal muscle mitochondria from control and patient 2 and in-gel activity staining for ATP synthase (complex V) activity.

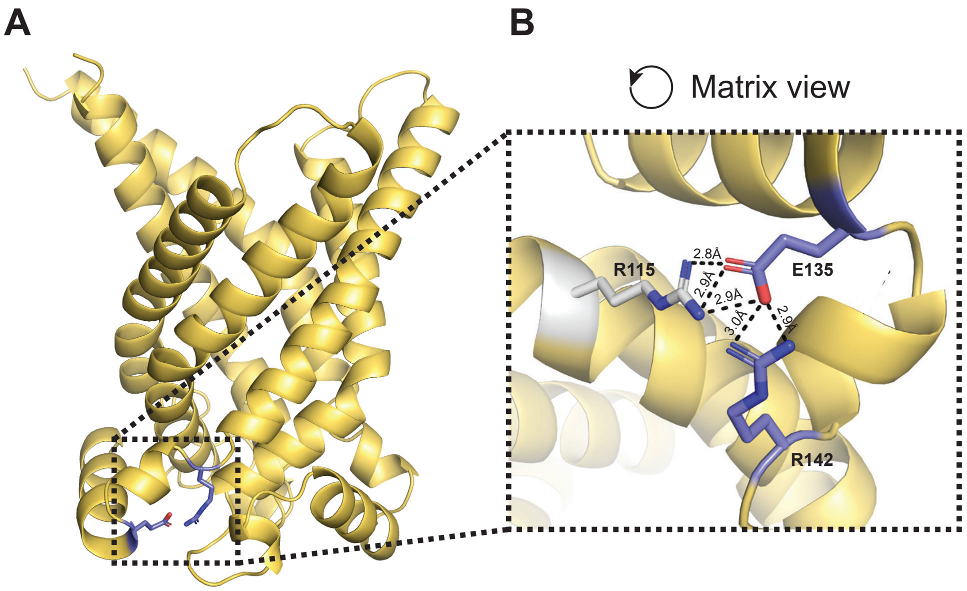

Fig. S2. E135 and R142 interact with each other in a structural prediction. (**A**) Prediction of murine SLC25A26 downloaded from the EMBL-EPI AlphaFold (34) database (UniProt accession Q5U680). Intermembrane space on top, and highlighted mutated residues facing the mitochondrial matrix. (**B**) Enlargement showing the sites E135 and R142 of two novel patient mutations (coloured in purple, oxygen in red, nitrogen blue), polar interactions and distances (dotted lines) and the interacting residue R115 (grey).

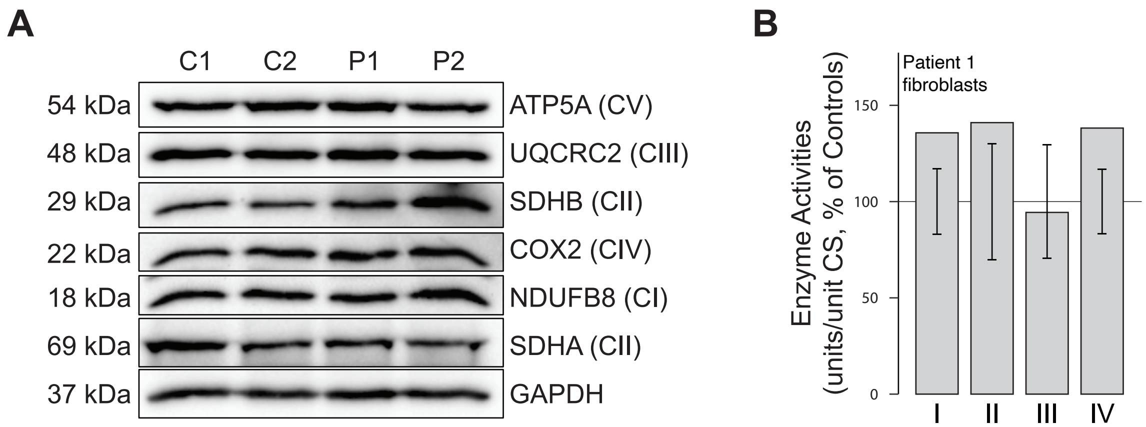

Fig. S3. Unaffected OXPHOS steady-state levels on denaturing PAGE in fibroblasts of patient 1. (**A**) Denaturing PAGE of total fibroblast extracts from Control (C1, C2) and patients (P1, P2), decorated with antibodies against mitochondrial respiratory chain subunits as indicated. (**B**) Isolated respiratory chain enzyme activities for NADH:ubiquinone oxidoreductase (I), succinate:ubiquinone oxidoreductase (II), ubiquinone:cytochrome c oxidoreductase (III) and cytochrome c oxidase (IV), relative to Controls (mean ± SD, n = 8).

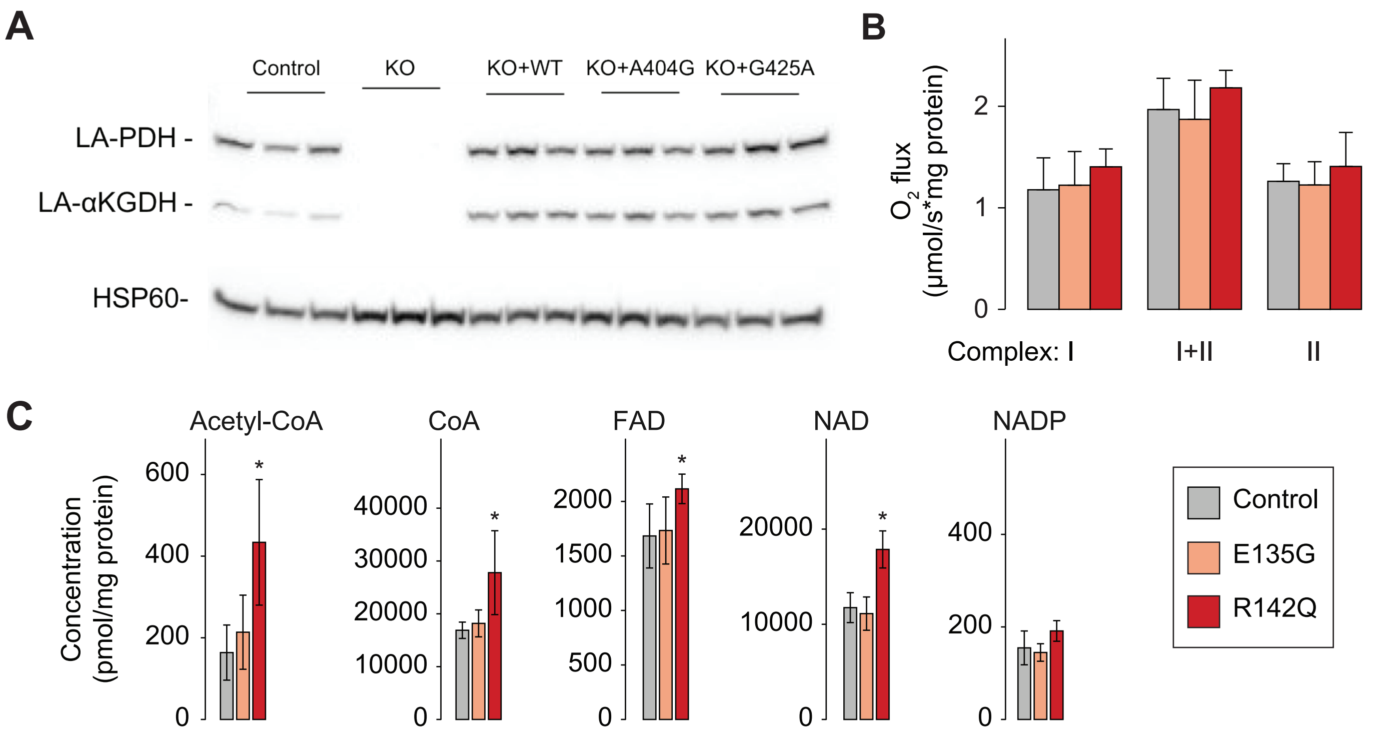

Fig. S4. Overexpression of human SLC25A26 variants in a mouse KO cell line. (**A**) Quantification of lipoic acid levels on pyruvate dehydrogenase (PDH) and α-ketoglutarate dehydrogenase (α-KGDH) by denaturing SDS-PAGE and Western blotting. KO: SLC25A26 knock-out cell line published in (33); KO+WT: Overexpression of human SLC25A26 in a KO background (n = 3). (**B**) Mitochondrial oxygen consumption quantified on an OROBOROS oxygraphy respirometer (n = 3). (**C**) Absolute quantification of indicated metabolites in total cell extracts. Control: Overexpressing human SLC25A26. E135G and R142Q are slc25a26 KO MEFs expressing either p.Glu135Gly or Arg142Gln, respectively. *P<0.05, unpaired two-sided Student's *t* test.

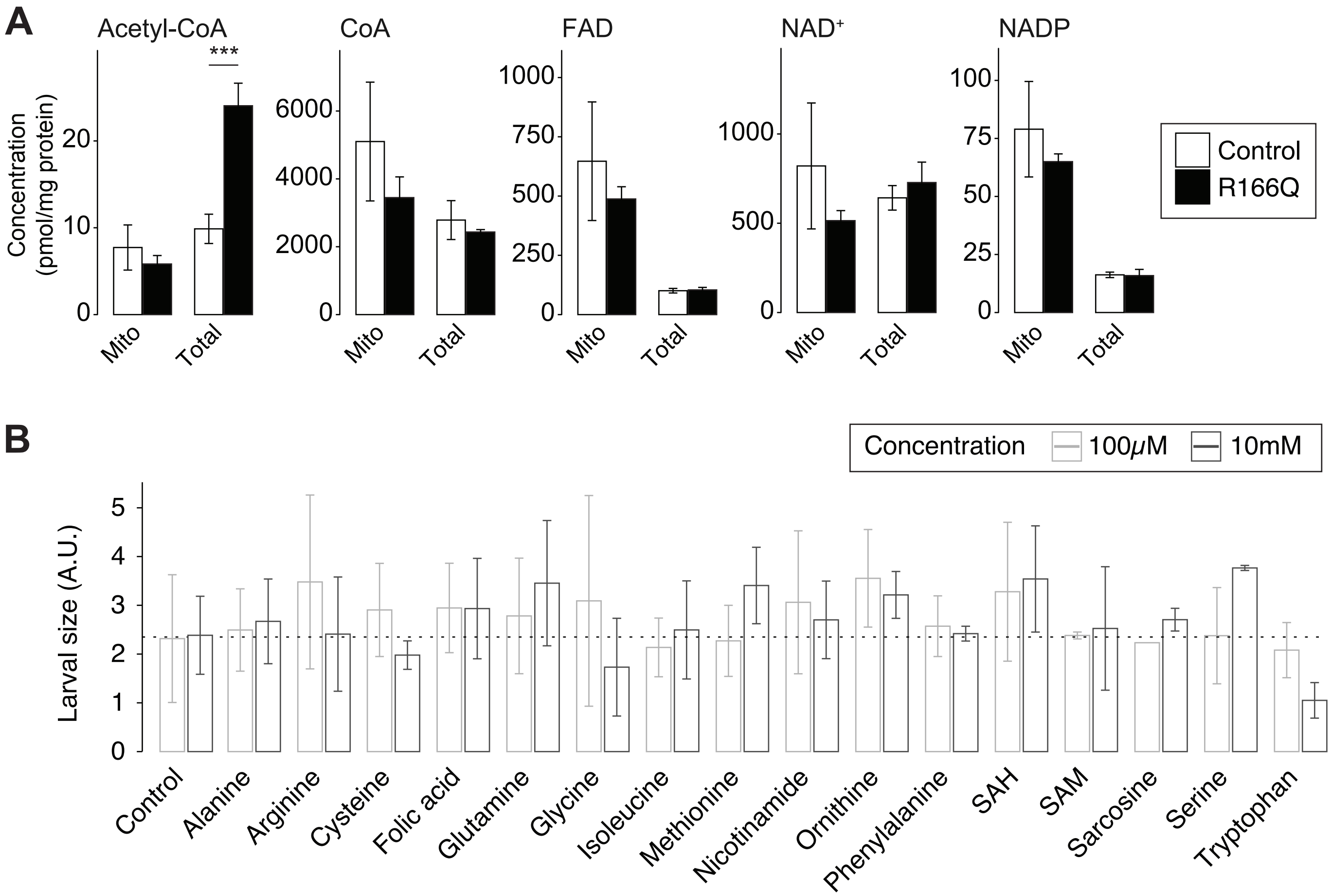

Fig. S5. Metabolic phenotyping in larval p.Arg166Gln Dm model. (**A**) Absolute levels of metabolites in fast mitochondrial enriched fractions or total larval extracts. R166Q are larvae homozygous for p.Arg166Gln. ***P < 0.001 with two-sided Student's t test (n = 3). (**B**) Size of larvae at 4 dae after developing on standard yeast-based food supplemented with metabolites at indicated concentration relative to water-treated control (n ≥ 10).
